# Improved Production of Recombinant Carboxylesterase FumDM by Co-Expressing Molecular Chaperones in *Pichia pastoris*

**DOI:** 10.3390/toxins15020156

**Published:** 2023-02-14

**Authors:** Lixiang Jiang, Xiao Guan, Hujun Liu, Xiaojiao Chang, Jing Sun, Changpo Sun, Chengcheng Zhao

**Affiliations:** 1 School of Health Science and Engineering, University of Shanghai for Science and Technology, Shanghai 200093, China; 2 Academy of National Food and Strategic Reserves Administration, Beijing 100037, China

**Keywords:** fumonisins, detoxification, carboxylesterase, chaperones

## Abstract

Fumonisins (FBs) are mycotoxins that threaten public health and food safety worldwide. Enzymatic degradation of Fumonisin B1 (FB_1_) through decarboxylation has attracted much attention, whereas application of FB_1_ carboxylesterase in detoxification requires more effective expression of the recombinant carboxylesterase. In this study, the carboxylesterase FumDM from *Sphingopyxis* sp. ASAG22 was codon-optimized and co-expressed with five different molecular chaperones (PDI, CPR5, ERO1, HAC1, and Bip) in order to improve the expression level of FumDM in *Pichia pastoris* (also known as *Komagataella phaffii*) GS115. The co-expression of different chaperones caused varying degrees of improvement in FumDM activity for FB_1_. The enzyme activities of recombinant strains over-expressing PDI and CPR5 reached the highest levels of 259.47 U/mL and 161.34 U/mL, 635% and 357% higher than the original enzyme activity, respectively. Transcriptomic analysis of the two recombinant strains in comparison with the control strain showed that the correct folding of proteins assisted by molecular chaperones played a key role in the improvement of FumDM expression and its enzyme activity. This study demonstrated that co-expression of carboxylesterase FumDM and folding chaperones was an efficient strategy and therefore might inspire new perspectives on the improvement of carboxylesterase for detoxification of FB_1_.

## 1. Introduction

The Food and Agriculture Organization of the United Nations (FAO) reports that about 25 percent of the world’s food crops are contaminated with mycotoxins every year, which can cause the loss of 1 billion tons of agricultural products [1]. Therefore, mycotoxins pose a very serious threat to food safety, food hygiene, and international trade [2]. Fumonisins (FBs), water-soluble secondary metabolites produced by several Fusarium species [3], are a group of widespread mycotoxins that mainly exist in maize and maize-based foods [4,5]. Fumonisin B1 (FB_1_) is found in the greatest proportion (about 70%) among FBs [6]. Moreover, FB_1_ also has neurotoxicity, immunotoxicity, organ and tissue toxicity, reproductive toxicity, and carcinogenicity effects in humans as well as in animals [7]. Due to its strong toxicity, the International Agency for Research on Cancer (IARC) has classified FB_1_ as a class 2B carcinogen [8]. Meanwhile, the United States Food and Drug Administration (FDA) and the European Commission have also formulated guidelines and regulations on FB_1_ for consumption [9]. Thus, reducing FB_1_ contamination efficiently is an urgent task in order to protect human and animal health away from their serious threat.

The biodegradation of FB_1_ has substantial potential applications in food and feed. Enzymatic degradation of FB_1_ has attracted much attention in recent years because of its advantages of substrate specificity, fast degradation rate to mycotoxin, excellent stability in complex matrices, and environmental friendliness [10]. During the enzymatic reaction process, the two tricarballylic acid (TCA) moieties, which are mainly responsible for FB_1_ toxicity, could be released by carboxylesterase [11]. Recently, several FB_1_ detoxification carboxylesterases and their responding coding genes were identified and expressed in different hosts. Carboxylesterase FumDSB from *Sphingomonadales* bacterium expressed in *Escherichia coli* (*E. coli*) [12] and the carboxylesterase FumD from *Sphingopyxis* sp. MTA144 expressed in *Pichia pastoris* (*P. pastoris*, also known as *Komagataella phaffii*) GS115 can degrade FB_1_ to hydrolyzed fumonisin B1 (HFB_1_) and thereby reduce the toxicity significantly [11]. A commercial enzyme-based feed additive containing fumonisin detoxification carboxylesterase was permitted to detoxify FB_1_ by the European Food Safety Authority (EFSA) [13]. However, only a few fumonisin detoxification carboxylesterases were expressed in *P. pastoris* and used in the preparation of feed additives because of their relatively low expression and activities. Therefore, more efforts should be made to promote the expression of fumonisin detoxification enzymes. 

The eukaryotic expression system of *P. pastoris* GS115 is becoming one of the most attractive platform for a plethora of exogenous expression recombinant proteins at present since it has many advantages over prokaryotes, such as a convenient cultivation method, rapid growth, easy genetic manipulation, and modification after translation [14]. Over-expression of recombinant genes may overwhelm the capacity of the endoplasmic reticulum (ER), which causes the accumulation of unfolded or misfolded proteins in the ER and induces the generation of the unfolded protein response (UPR) and ER degradation pathway (ERAD), consequently resulting in a reduction in the ability to produce and secrete recombinant proteins [15]. Importantly, many studies have suggested that molecular chaperones may be able to significantly enhance the de novo folding of proteins at the ribosome and their translocation across membranes with other chaperone systems [16,17]. In this regard, the most widely understood is the mediation of two folding enzymes, protein disulfide isomerase (PDI) and peptidyl-prolyl cis-trans isomerase (PPI). PDI can catalyze the exchange reaction between sulfhydryl and disulfide bonds, promoting the formation, isomerization, or reduction of disulfide bonds in proteins [18], and almost more than 30% of proteins in the ER need PDI to promote the formation of disulfide bonds during the folding process [19]. PPI stabilize the twisted amide transition state through a non-covalent bond and catalyzes the mutual transformation of cis and trans rotators between peptidylprolyl groups. CPR5, a member of the cyclophilin family belonging to PPI, is able to catalyze the initial process of protein folding and the rate-limiting step in the process of oligomer rearrangement [20,21]. HAC1 plays a key role in activating and maintaining the UPR pathway and promotes the expression of UPR-related proteins such as ER oxidoreductase (ERO1) and Bip [22]. ERO1 can oxidize PDI from its reduced state to its oxidized state [23]. Bip, a heavy chain binding protein, can bind to newly synthesized misfolded polypeptides and prevent their aggregation in the ER [24]. Therefore, co-expressing molecular chaperones with heterologous proteins is an attractive strategy for the development of efficient protein expression systems.

This work aimed to engineer the protein folding system in *P. pastoris* for the improvement of fumonisin carboxylesterases. In this study, FB_1_ carboxylesterase from *Sphingopyxis* sp. ASAG22 was heterologously expressed in *P. pastoris* GS115 with the combined use of codon optimization and molecular chaperone co-expression. The codon-optimized FumDM was co-expressed with five different molecular chaperones (PDI, CPR5, ERO1, HAC1, and Bip), and the recombinant strains overexpressing PDI and CPR5 were selected according to expression and enzyme activity. Afterwards, transcriptomic analysis was performed to investigate the role of molecular chaperones in promoting the expression of FumDM, thereby providing a reference for further improvement.

## 2. Results

### 2.1. Construction and Selection of Recombinant P. pastoris Expressing Carboxylesterase FumDO/FumDM

*FumDO* (original carboxylesterase gene without codon-optimization) and *FumDM* (codon-optimized carboxylesterase gene) all have the same open reading frame of 1623 bp, encoding a protein of 541 amino acids. The molecular mass and pI of FumDO/FumDM were predicted to be 57.5 kDa and 9.42, respectively.

**Figure 1:**
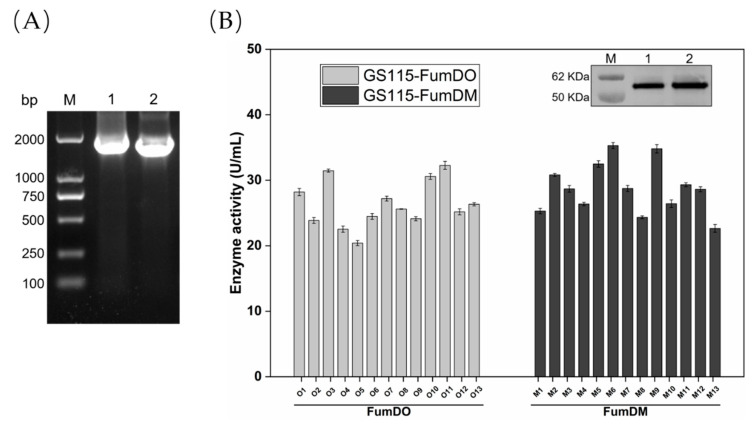
Construction and expression of recombinant strains GS115-FumDO and GS115-FumDM. (**A**) Electrophoresis pattern of PCR products for FumDO and FumDM. Lane M, DL 2000 DNA Marker (2000, 1000, 750, 500, 250, and 100 bp); Lanes 1–2, PCR results for the purpose of FumDO and FumDM, respectively. (**B**) Enzyme activity of recombinant strains GS115-FumDO/GS115-FumDM and western blot analysis (Lane M, molecular mass marker. Lane 1, the recombinant strains GS115-FumDO. Lane 2, the recombinant strains GS115-FumDM).

As shown in Figure 1A, FumDO and FumDM were cloned and inserted into pPIC9K. Correct construction was confirmed by sequencing. Afterwards, plasmids linearized by *Sal*I were transformed into *P. pastoris* GS115 by electroporation.

O1-O13 and M1-M13 denote the 13 GS115-FumDO and 13 GS115-FumDM *P. pastoris* positive transformants obtained, respectively. These recombinant strains were screened for FB_1_ detoxification activities. The highest enzyme activities of GS115-FumDO and GS115-FumDM were 32.27 U/mL and 35.30 U/mL, respectively, achieved by O11 and M6 in the supernatant of the medium cultured for 5 days (Figure 1B). It is also observed that the enzyme activities of O11 and M6 increased during the fermentation process and showed significant differences after 5 days of induction (Figure S1). In addition, there was no activity detected that harbors an empty plasmid, pPIC9K, whether in the medium supernatant or in the intracellular supernatant. Therefore, M6 was selected for co-expression with molecular chaperones.

### 2.2. Construction and Selection of Recombinant P. pastoris Co-Expressing Carboxylesterase FumDM and Molecular Chaperones

**Figure 2:**
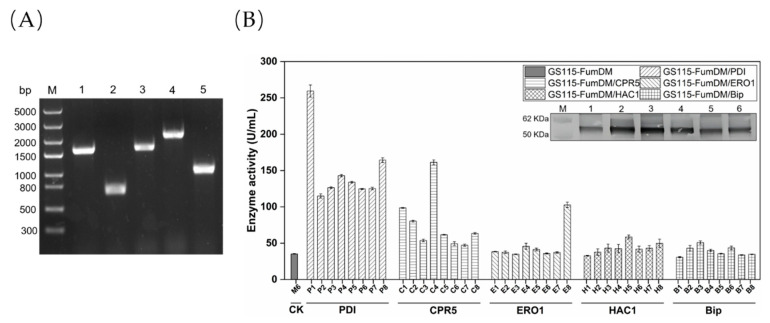
Construction and expression of recombinant strains co-expressing of molecular chaperones (**A**) Electrophoresis pattern of PCR products for five molecular chaperone genes. Lane M, Trans 5k DNA Marker (5000, 3000, 2000, 1500, 1000, 800, 500, and 300 bp); Lanes 1–5, PCR results for the purpose of *PDI*, *CPR5*, *ERO1*, *Bip* and *HAC1*, respectively. (**B**) Enzyme activity of recombinant strains co-expressing of molecular chaperones and western blot analysis (Lane M, molecular mass marker. Lane 1, the FumDM without molecular chaperone co-expression. Lanes 2–6, the FumDM with PDI, CPR5, ERO1, HAC1, and Bip co-expression, respectively).

To investigate the effect of molecular chaperones on promoting expression of FumDM in *P. pastoris*, five molecular chaperone (PDI, CPR5, ERO1, Bip, and HAC1) genes were cloned from the *P. pastoris* GS115 genome (Figure 2A), inserted into the pPICZA vector to obtain the recombinant plasmids (pPICZA-PDI, pPICZA-CPR5, pPICZA-ERO1, pPICZA-Bip and pPICZA-HAC1), and then transformed into strain M6. The recombinant *P. pastoris* co-expressing carboxylesterase FumDM and molecular chaperones were screened on YPD plates containing 100 µg/mL Zeocin and verified by PCR. P1–P8, C1–C8, E1–E8, H1–H8, and B1–B8 denote the eight GS115-FumDM/PDI, eight GS115-FumDM/CPR5, eight GS115-FumDM/ERO1, eight GS115-FumDM/HAC1, and eight GS115-FumDM/Bip *P. pastoris* positive transformants obtained, respectively.

Given that molecular chaperones play different roles in a wide variety of cellular processes, it was crucial to select the optimal chaperones from five candidates. As shown in Figure 2B, the highest enzyme activities of 259.47 U/mL were achieved in the transformant P1 after 5 days of cultivation, followed by the transformants C4 (161.34 U/mL), E8 (102.66 U/mL), H5 (58.50 U/mL), B3 (50.86 U/mL), and control M6 (35.30 U/mL). When the enzyme activity was monitored at different time points from the 1st day to the 5th day, FumDM activities in P1 and C4 strains (co-expressing PDI and CPR5, respectively) were significantly higher than those in other strains (Figure S2). Likewise, these selected transformants (P1, C4, E8, H5, B3, and M6) showed different expression levels of recombinant FumDM (57 kDa) as determined by western blot. Based on these results, the recombinant *P. pastoris* strains P1 and C4 were selected for further investigation.

### 2.3. Transcriptomic Analysis

#### 2.3.1. Sequencing, Evaluation, and Annotation of the Transcriptomic Profile

Since molecular chaperones PDI and CPR5 could significantly promote the expression of carboxylesterase FumDM, RNA sequencing was conducted to profile the transcriptome changes in the M6, C4, and P1 strains. The sequencing samples were named CK, CP, and PD to represent the recombinant strains M6, C4, and P1, respectively. The CK sample served as the control group, the CP and PD samples were the experimental groups, and each group performed three parallel experiments at the same time. The two experimental groups and the control group were compared to obtain CK vs CP and CK vs PD comparison groups. To our knowledge, this is the first transcriptional reaction profile of FumDM co-expression with CPR5 and PDI in *P. pastoris*.

**Figure 3:**
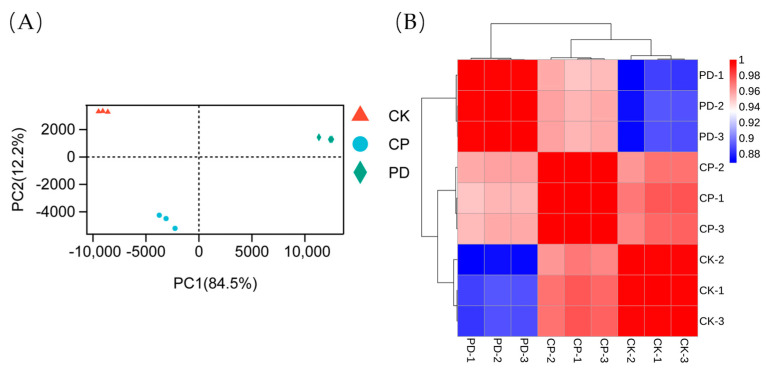
Gene expression in different groups. (**A**) Principal component analysis of samples. The color and shape of the scatter plot represented the grouping of samples. CK represented the FumDM sample without molecular chaperone co-expression. CP and PD were used to denote the FumDM co-expression with CPR5 and PDI, respectively. (**B**) Correlation analysis of comparison groups. CK-1, CK-2, and CK-3 were parallel samples, and the rest were numbered similarly. The color indicated the level of correlation for each sample, from low (blue) to high (red).

**Figure 4:**
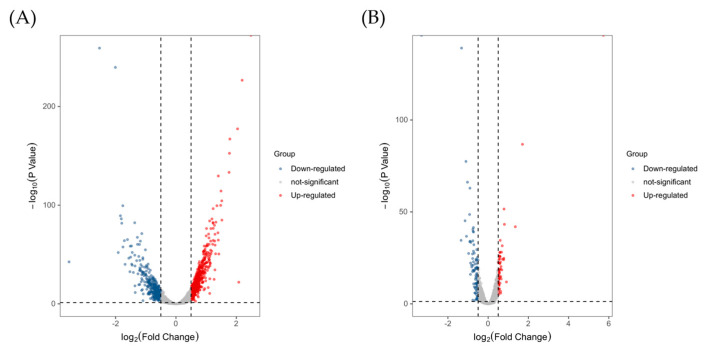
Volcano plots of DEGs in different groups. (**A**) The DEGs in the PD group. (**B**) The DEGs in the CP group.

After transcriptome sequencing and processing, a total of 56.47 Gb of clean data was obtained for nine samples. The experimental results were listed in Table S1. Data ranging from 5.8 Gb to 7.7 Gb were generated for each sample, and the base sequencing Q30 (%) was greater than 93.59 %, indicating that the sequencing quality was relatively high. Principal component analysis (PCA) and Pearson correlation analysis showed that the three groups were differentiated from each other with good biological repeatability (Figure 3A,B), making them suitable for further bioinformatics analysis. The volcano plots (Figure 4) were applied for visualizing distributions of differentially expressed genes (DEGs), illustrating that there were obvious differences in gene expression among the three strains. Compared with the control group, 657 DEGs were screened in the PD group (including 393 up-regulated and 264 down-regulated genes) and 91 DEGs were found in the CP group (including 23 up-regulated and 68 down-regulated genes).

#### 2.3.2. Functional Classification of DEGs and Pathway Analysis

The DEGs were associated with a wide range of regulatory and metabolic processes. To further assign the functions, metabolic pathways, and interrelationships between the DEGs, the DEGs were mined using GO functional analysis and KEGG pathway analysis. GO enrichment analysis (Tables S2 and S3) revealed the top 20 functional annotations of the up-regulated DEGs. The most significantly enriched GO terms in two groups were related to isomerization in protein folding, lipid metabolism, and response to stress. For example, DEGs in the PD group were assigned to protein disulfide isomerase activity, oxidoreductase activity, and unfolded protein binding in the molecular functions domain and protein folding, protein refolding, the lipid biosynthetic process, the unsaturated fatty acid biosynthetic process, and response to stimulus in the biological processes domain. In the CP group, highly-enriched functions in the molecular functions domain were peptidyl-prolyl cis-trans isomerase activity, kinase activity, and cellular response to oxidative stress, phospholipid translocation, fatty acid beta-oxidation in the biological processes domain. All other functions in two groups were moderately enriched.

**Figure 5:**
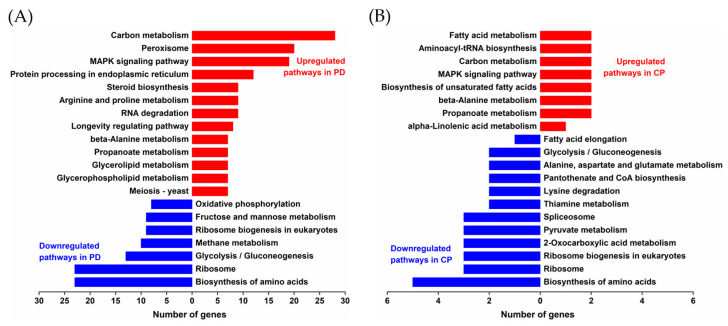
KEGG analysis of the top 20 differentially regulated pathways. (**A**) Differentially regulated pathways in the PD group. (**B**) Differentially regulated pathways in the CP group.

The top 20 metabolic pathways were divided into two groups and annotated by KEGG pathway analysis (Figure 5). In the PD group, the up-regulated pathways were primarily involved in carbon metabolism, peroxisomes, the MAPK signaling pathway, protein processing in the ER, and steroid biosynthesis. In the CP group, the up-regulated pathways were mainly related to fatty acid metabolism, aminoacyl-tRNA biosynthesis, carbon metabolism, the MAPK signaling pathway, and the biosynthesis of unsaturated fatty acids. Notably, biosynthesis of amino acids and ribosomes were two significant down-regulated pathways that appeared in both groups.

#### 2.3.3. Validation of DEGs by qRT-PCR

**Figure 6:**
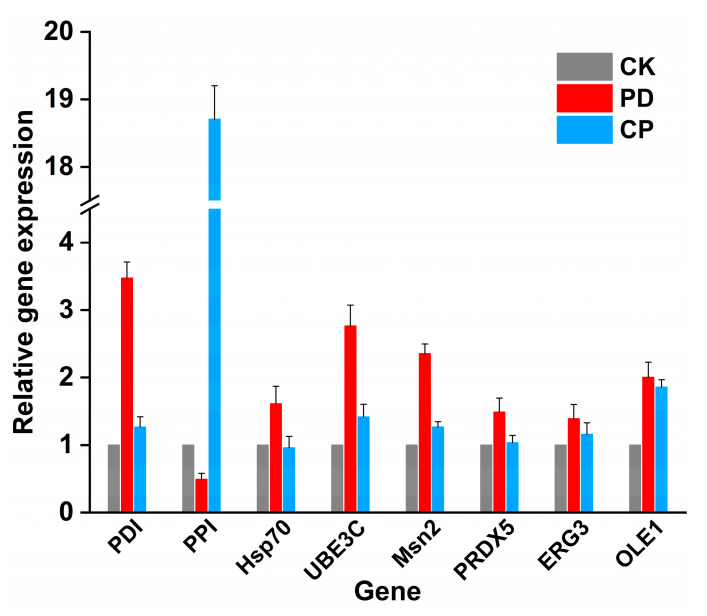
Relative expression of DEGs in the two groups was verified by qRT-PCR. The horizontal coordinate was the gene name, while the vertical coordinate was the difference in ploidy of the experimental group’s genes compared with those of the control group. A difference multiplier greater than 1 indicated upregulation, and vice versa for downregulation. The expression of DEGs in PD and CP groups was illustrated by the red and blue bars. Each column represents the mean of three replicates, and the results are presented as the mean ± standard error.

Eight DEGs, which were mainly related to protein folding, transport, and hydrolysis, UPR, stress response, and lipid metabolism, were selected to validate the expression levels of the genes by qRT-PCR (Figure 6). Two folding enzymes, protein disulfide isomerase (*PDI*) and peptidyl-prolyl cis-trans isomerase (*PPI*) were dramatically up-regulated in the PD and the CP group, respectively, further confirming the over-expression of PDI and CPR5. The expression levels of ubiquitin-protein ligase E3 C (*UBE3C*), zinc finger protein (*Msn2*), and delta(9) fatty acid desaturase (*OLE1*) were all up-regulated to different degrees in the PD and CP groups. However, heat shock protein 70 (*Hsp70*), thiol-specific peroxiredoxin (*PRDX5*), and C-5 sterol desaturase (*ERG3*) showed up-regulation in the PD group but were moderately expressed in the CP group. These qRT-PCR results showed good agreement with RNA-seq results, indicating the results of transcriptomic analysis were stable and reliable.

## 3. Discussion

*Sphingopyxis* species have been widely used in the degradation of environmental pollutants such as toxins, petroleum, and pesticides because of their various hydrolases and transport enzymes [25]. For example, carboxylesterases from *Sphingopyxis* sp. were used as excellent degrading enzymes of residual pesticides to hydrolyze carbaryl, carbofuran, metaphos, and bifenthrin [26]. Recently, fumonisin carboxylesterases from *Sphingopyxis* sp. have gained widespread attention due to their potential in FB_1_ degradation. So far, many carboxylesterases have been isolated and heterologously expressed in different host systems. However, the application of carboxylesterase was limited by its low expression level. Co-expression of target genes with molecular chaperones has been established as an efficient way to promote the yield of heterologous proteins [27]. Therefore, in this study, codon-optimized carboxylesterase FumDM was co-expressed with molecular chaperones in an attempt to improve the expression level of FumDM in *P. pastoris*. Theoretically, PDI plays a key role in the formation and rearrangement of disulfide bonds in newly folded proteins [27]. CPR5 helps protein folding and assembly by catalyzing the cis-trans isomerization of peptides containing peptidyl-prolyl [28]. Whereas, HAC1 is a transcription factor and a master regulator of the UPR. HAC1 could orchestrate a complex transcriptional response, including transcription of ER-resident chaperones like ERO1 and Bip, to mitigate ER stress [29]. Our study showed that a codon-optimized carboxylesterase, FumDM, was expressed in *P. pastoris* as a 57 kDa secreted protein, which was confirmed by western blot, and the enzyme activity of FumDM was 35.30 U/mL at the shake-flask fermentation. Compared with the original activity, the enzyme activities of FumDM in the supernatant of the recombinant strains co-expressing PDI, CPR5, ERO1, HAC1, and Bip were increased by 635%, 357%, 191%, 66%, and 44%, respectively. Similar results were also reported in other studies. For instance, the enzyme activity of the rBmAChE2 with four potential intramolecular disulfide bonds was increased by almost 5 times after co-expression with PDI [30]. In addition, co-expression of HAC1, PDI, and ERO1 could increase the activity of mannanase ManA in the supernatant by 26%, 20%, and 15%, respectively [31]. In the present study, co-expression of different chaperones led to varying degrees of improvement in the enzyme activity of FumDM, indicating that improved folding of recombinant FumDM might bring about increased protein expression and correspondingly increased activity. Particularly, the considerable enhancement of FumDM caused by PDI and CPR5 co-expression was probably due to the amino acid sequence of FumDM, which contained 5 cysteines and 42 prolines.

Transcriptome profiles of two selected strains, P1 and C4, in comparison with the control strain M6, were analyzed to further clarify the regulation of molecular chaperones PDI and CPR5 at the level of gene transcription. GO functional analysis showed that, in the PD group, the DEGs related to protein disulfide isomerase, lipid biosynthetic processes, and response to stimulus were significantly up-regulated; in the CP group, the DEGs related to peptidyl-prolyl cis-trans isomerase, sterol isomerase, and the cellular response to oxidative stress were significantly up-regulated. KEGG pathway analysis revealed that the DEGs related to the biosynthesis of amino acids and ribosomes were significantly down-regulated in both groups. It was summarized that the DEGs associated with isomerization in protein folding, lipid metabolism, response to stress, and ribosome played impressive roles in the improvement of expression and enzyme activity of FumDM.

It has been reported that the protein folding process is rate-limited by the isomerization of covalent bonds and requires the involvement of folding catalysts [21,32]. In this study, two protein-folding catalysts, PDI and PPI, were significantly up-regulated in the PD and CP groups, respectively. PDI can not only oxidize cysteine in new polypeptides to form disulfide bonds, but also form a central cavity with the dimer in the form of oxidation and bind the unfolded substrate so that the substrate can temporarily remain in the limited hydrophobic space for folding or oxidative folding [33]. Over-expression of PDI may form more central cavities and larger hydrophobic spaces to combine more unfolded substrates and prevent the accumulation of unfolded proteins in the ER from causing ER stress. In the PD group, over-expression of PDI might catalyze the formation of correct disulfide bonds between cysteine residues in FumDM and thus promote increased FumDM expression. CPR5, a cyclophilic protein, has peptidyl-prolyl cis-trans isomerase activity, which catalyzes the isomerization of peptide bonds from trans- to cis-form at proline residues, fundamentally changing the local and long-range structures in proteins [34]. Proline residues have unique roles in protein folding, structure, and function. Besides, proline can locally interact with aromatic amino acids to stabilize cis-amide bonds [35,36]. 42 prolines and 47 aromatic amino acids (12 Tyrosine, 23 Phenylalanine, and 12 Tryptophan) were found in FumDM according to the protein sequence. Thus, we deduced that over-expression of CPR5 would facilitate the isomerization of proline-rich FumDM, which accelerated the folding rate of FumDM. In addition, transcription factor HAC1 and ubiquitin protein ligase E3 C were significantly up-regulated in two groups, indicating the regulation of the UPR and ERAD pathways. The difference was that some heat shock proteins (Hsp40 and Hsp70) participating in protein folding were up-regulated in the PD group but moderately expressed in the CP group. The fact that the protein folding-related gene in the PD group was richer than the CP group is not unreasonable, considering that PDI is a multifunctional protein resident in the ER lumen and the enzyme activity of FumDM in strain P1 was higher than that in strain C4. In summary, the genes related to protein folding were significantly up-regulated after co-expression with PDI and CPR5, and the over-expression of protein-folding catalysts would promote the correct folding of proteins and relieve ER stress, resulting in increased production of recombinant FumDM.

Protein synthesis and transmembrane transport are inevitably linked to the membrane. Hence, many DEGs would be expected to be associated with membrane structures and functions. The RNA-seq data highlighted differential expression of cell membrane constituents, such as C-8 sterol isomerase and delta (9) fatty acid desaturase, which were up-regulated in two groups; fatty acid eloncase 3, which was down-regulated in two groups; and C-5 sterol desaturase and C-4 methyl sterol oxidase, which were up-regulated in the PD group. The up-regulation of ergosterol-related genes would promote the combination with phospholipids to stabilize membrane structure and thus enhance the ability of membrane structure fluidity, permeability, membrane binding enzyme activity and material transport [37]. The decrease of long-chain fatty acids and the increase of unsaturated fatty acids could significantly improve the fluidity of the cell membrane and protect cells from various external pressures [38]. In our study, genes involved in ergosterol biosynthesis were up-regulated, and the content of short-chain fatty acids and unsaturated fatty acids was increased, indicating that cells could improve membrane fluidity or permeability by regulating the composition and content of fatty acids, which was conducive to the synthesis and secretion of FumDM.

Changes in environmental conditions will disturb the redox balance of cells and cause oxidative stress. Recombinant strains can respond to oxidative stress through enzymatic and non-enzymatic defense systems to avoid or mitigate the impact of this phenomenon on protein folding and activity. Zinc finger protein, nitroreductase, and cell division control protein were up-regulated in two groups; glutathione S-transferase and thiol-specific peroxiredoxin were up-regulated in the PD group. The zinc finger protein is able to regulate gene expression and stress response together with several transcription factors (AtfB, SrrA, and AP-1) [39,40]. The up-regulation of nitroreductase and thiol-specific peroxiredoxin could alleviate the oxidative damage caused by hydrogen peroxide and regulate intracellular redox homeostasis and cell elasticity [41]. The up-regulation of glutathione S-transferase could catalyze the combination of glutathione and a variety of foreign compounds and increase the level of glutathione-based antioxidative activity [42]. Cell division control protein transcription increases as cells begin to repair stress-induced damage, which helps regulate cell polar growth and restore retarded cell cycle progression [43]. Our results showed that oxidative stress kinase and the non-enzyme defense system activated the defense function against stress reactiond, indirectly improved the viability of recombinant strains, and provided a stable environment for FumDM.

Interestingly, a large number of proteins involved in ribosome and ribosome biogenesis in eukaryotes showed a downward trend in our study. Most of these proteins were contributing to the ribosomal proteins (the small (40S) ribosomal proteins and the large (60S) ribosomal protein families) and the assembly and nuclear export of ribosomal subunits. Studies have shown that cells tend to inhibit the translation system to improve ribosome efficiency and save energy under the conditions of unfolded protein pressure [44] and excessive expression of foreign proteins [45]. It has been reported that increased expression of target proteins was accompanied by decreased expression of ribosomal proteins in recombinant strains co-expressing HAC1 [46,47]. Ribosome biogenesis in eukaryotes also reveals a significant correlation between ribosomal protein gene deletion and the reduction of ER stress [48]. Consistent with this, the down-regulation of genes involved in ribosome biogenesis in our study might slow down translation and thereby prevent UPR and improve the efficiency of FumDM synthesis.

The present results demonstrated that engineering protein folding by co-expressing folding-related molecular chaperones was an effective strategy for the improvement of carboxylesterase that could be used for reducing FB_1_. FumDM might be a potential feed additive for detoxification of FB_1_ in feedstuffs, but further enhancement of expression and large-scale production of FumDM are required for industrial applications.

## 4. Conclusions

In this study, a carboxylesterase, FumDM, from *Sphingopyxis* sp. ASAG22 was codon-optimized and then co-expressed with five different molecular chaperones in *P. pastoris* GS115. Over-expression of PDI, CPR5, ERO1, HAC1, and Bip could enhance the expression of FumDM, and the highest levels of these enzyme activities for FB_1_ were 259.47 U/mL, 161.34 U/mL, 102.66 U/mL, 58.50 U/mL, and 50.86 U/mL, respectively. Compared with the original enzyme activity, the FumDM activities of the five strains co-expressing molecular chaperones were increased by 635%, 357%, 191%, 66%, and 44%, respectively. Transcriptome profiling of two recombinant strains (over-expressing PDI and CPR5, respectively) revealed that molecular chaperones PDI and CPR5 could improve FumDM production by facilitating the correct folding and assembly of proteins. Moreover, cellular changes in lipid metabolism, stress response, and ribosome biosynthesis might alleviate ER stress and thus contribute to the increased expression of recombinant FumDM. This study proposed effective expression of the recombinant carboxylesterase FumDM in *P. pastoris* and therefore made FumDM a promising candidate for the detoxification of FBs in food and feed.

## 5. Materials and Methods

### 5.1. Strains, Plasmids, and Medium

The strains, plasmids, and primers used in this study are listed in Table 1. *E. coli* DH5α, purchased from Takara (Dalian, China), was used for plasmid proliferation. *P. pastoris* strain GS115, two yeast expression vectors, pPIC9K and pPICZA, as well as all restriction enzymes (*Eco*RI, *Not*I, *Xho*I, *Sal*I, *Sac*I, and *Pme*I) were purchased from Invitrogen (Carlsbad, CA, USA).

Luria-Bertani (LB) medium contained 10 g/L sodium chloride, 10 g/L tryptone, and 5 g/L yeast extract. The yeast extract peptone dextrose (YPD) medium contained 20 g/L glucose, 20 g/L tryptone, and 10 g/L yeast extract. The minimal dextrose (MD) medium contained 13.4 g/L yeast nitrogen base (YNB), 4 × 10^−4^ g/L biotin, and 20 g/L glucose. The buffered glycerol-complex (BMGY) medium contained 20 g/L tryptone, 10 g/L yeast extract, 13.4 g/L yeast nitrogen base (YNB), 4 × 10^−4^ g/L biotin, 100 mmol/L potassium phosphate buffer (pH 6.0), and 10 g/L glycerol. Buffered methanol-complex (BMMY) medium contained 20 g/L tryptone, 10 g/L yeast extract, 13.4 g/L yeast nitrogen base (YNB), 4 × 10^−4^ g/L biotin, 100 mmol/L potassium phosphate buffer (pH 6.0), and 5 g/L methanol.

### 5.2. Construction of Recombinant Strains

The carboxylesterase that could degrade FB_1_ was previously amplified from *Sphingopyxis* sp. ASAG22 and named *FumDO* in our laboratory. The codon-optimized carboxylesterase gene *FumDM* was synthesized by Sangon Biotech (Shanghai) Co., Ltd. *FumDO* and *FumDM* were amplified via polymerase chain reaction (PCR; C1000 Touch™, Bio-Rad, Hercules, CA, USA) with primers *FumDO*-F/*FumDO*-R and *FumDM*-F/*FumDM*-R, respectively. The plasmid pPIC9K was digested with two restriction enzymes (*Eco*RI and *Not*I) and purified by the Omega Cycle-Pure Kit. The PCR products were purified using the AxyPrep Gel Recovery Kit, ligated to the pPIC9K vector by the In-Fusion HD Cloning Kit (Takara, Dalian, China), and then transformed into the chemically competent cells of *E. coli* DH5α. The recombinant plasmids pPIC9K-*FumDO* and pPIC9K-*FumDM* were verified by sequencing, linearized with the restrictive endonuclease *Sal*I, and purified by the Omega Cycle-Pure Kit. The linearized plasmids were transformed into *P. pastoris* GS115 competent cells by electroporation for 5 ms at 2.0 kV (GenePulser Xcell™, Bio-Rad, Hercules, CA, USA), and then the cells were cultured for 2–3 h at 30 °C. Finally, the cultured cells (200 μL) were uniformly coated on minimal dextrose (MD) medium, and the plates were incubated at 30 °C for 2–5 days. All transformants on MD medium were suspended in sterile water and screened on YPD solid medium containing different concentrations of G418. The strains were cultured in shake flasks, and cells from overnight cultures were collected to extract genome DNA for PCR identification and sequencing verification.

The genes coding for molecular chaperones PDI (AJ302014.1), CPR5 (XM_002489874.1), ERO1 (XM_002489600.1), HAC1 (XM_002489994.1), and Bip (XM_002490982.1) were amplified with primer pairs *PDI*-F/*PDI*-R, *CPR5*-F/*CPR5*-R, *ERO1*-F/*ERO1*-R, *HAC1*-F/*HAC1*-R, and *Bip*-F/*Bip*-R, respectively, using *P. pastoris* GS115 genome as templates. The PCR products of PDI, CPR5, and HAC1 were purified, digested with *Eco*RI and *Xho*I, and then inserted into the plasmid pPICZA, which had been digested with the same restriction enzymes. The PCR products of ERO1 and Bip were purified and then ligated to the *Eco*RI/*Xho*I-digested pPICZA plasmid by the In-Fusion Cloning Kit. These recombinant plasmids were transformed into the chemically competent *E. coli* DH5α cells. After verification by sequencing, the recombinant plasmid pPICZA-*ERO1* was linearized with *Pme*I, and the other recombinant plasmids were linearized with *Sac*I to obtain the linearized plasmids (pPICZA-*PDI*, pPICZA-*CPR5*, pPICZA-*HAC1*, pPICZA-*ERO1,* and pPICZA-*Bip*). These linearized plasmids were transferred into GS115-FumDM-competent cells by electroporation under the same conditions screened on the YPD solid medium containing 100 µg/mL Zeocin.

### 5.3. Screening and Cultivation of Strains

All the *E. coli* DH5α strains were cultured in LB medium at 37 °C. When necessary, the medium was supplemented with 50 µg/mL Kanamycin or 25 µg/mL Zeocin for the selection of transformants containing plasmids.

All recombinant *P. pastoris* positive strains were picked out and cultured in flasks containing YPD medium at 30 °C and 220 rpm for 24 h. Then the cultures were inoculated in BMGY medium with an inoculum volume of 0.5% and incubated in a rotary shaker incubator at 29 °C and 240 rpm for 12 h. When the cultures reached an OD_600_ = 2–6, cells were harvested by centrifuging at 3000× *g* for 5 min at room temperature and resuspended in fresh BMMY medium. A final concentration of 0.5% methanol was supplemented every 24 h to maintain induction. After 5 days of induction, the enzyme activity of FumDO/FumDM and the protein expression of the cultures were analyzed.

### 5.4. Analysis of Protein Expression

The fermentation broth was centrifuged at 13,000× *g* to separate the supernatants and cell pellets. Protein expression in the supernatants was analyzed by western blot. Total protein was transferred from an SDS-PAGE gel (Thermo Fisher Scientific, Waltham, MA, USA) to a polyvinylidene difluoride (PVDF) membrane (Bio-Rad, Hercules, CA, USA) using electrophoresis apparatus (Bio-Rad, Hercules, CA, USA) at 100 V for 3 h. The PVDF membrane was blocked with 5% skim milk for 2 h with gentle shaking at room temperature (RT, 25 °C) before being incubated with 1:2000 diluted anti-FumDM primary antibody overnight at 4 °C. After washing, the blot was incubated with alkaline phosphatase-conjugated anti-rabbit IgG (Beyotime, Shanghai, China) at 30 °C for 1 h. Finally, the blot was developed using BCIP/NBT Alkaline Phosphatase Color Development Kit (Beyotime, China) for 1 h in the dark and analyzed with the ChemiDoc^TM^ MP Imaging System (Bio-Rad, Hercules, CA, USA).

### 5.5. Enzymatic Activity Assay by High Performance Liquid Chromatography

FB_1_ degradation by FumDO or FumDM was performed using an appropriate amount of diluted enzyme solution in 50 mmol/L sodium phosphate buffer (pH 6.5) containing FB_1_ (5 µg/mL) at 40 °C for 10 min, and then the mixture was boiled for 10 min to terminate the reaction. One unit (U) of carboxylesterase activity was defined as the amount of enzyme required to degrade 1 µg FB_1_ per minute at 40 °C and pH 6.5.

FB_1_ detoxification activities of FumDO/FumDM in this study were detected by high-performance liquid chromatography (HPLC), which was performed on a Waters Alliance e2695 HPLC system with a 2475 fluorescence detector (Waters Co., Ltd., Milford, MA, USA). In order to add fluorescence detective signals onto FB_1_, samples were derived by mixing 100 µL of enzyme reaction mixture with 400 µL of 50% acetonitrile aqueous solution and 500 µL of a o-phthaldialdehyde (OPA) derivative solution. Then, samples were filtered through a 0.22 µm nylon filter and stored in sampler vials for injection within 2 min. Separation was performed on a C18 column (5 µm particle size, 250 × 4.6 mm X-bridge, Waters Co., Ltd., Milford, MA, USA) with a flow rate of 1.0 mL/min at 40 °C and the injection volume was set at 50 µL. All samples were analyzed at an excitation wavelength of 335 nm and an emission wavelength of 440 nm. The mobile phase consisted of 0.1 mol/L ammonium formate—formic acid aqueous solution (A) and 100% chromatographic grade methanol (B). The gradient was as follows: 0.00 min 30% A, 5.00 min 28% A, 6.00 min 25% A, 11.00 min 22% A, 11.10 min 30% A, and 16.00 min 30% A. Waters ChemStation was employed for the LC system to acquire and analyze chromatographic data. The degradation rate of FB_1_ was calculated using the following formula: (1—concentration of FB_1_ residual/concentration of FB_1_ original) × 100%.

### 5.6. Transcriptome Analysis

Strains were cultivated in the BMMY medium for 5 days, and then cells were collected and rapidly frozen in liquid nitrogen. Thereafter, the RNA libraries were constructed, and RNA sequencing was performed on the Illumina platform at Biomarker (BMK) Technologies Co., Ltd. (Beijing, China). Transcriptome analysis of sequencing samples was performed using BMKCloud (www.biocloud.net, accessed on 5 September 2022). The genomic DNA of *Komagataella phaffii* GS115 was used as a reference for gene annotation. The expression levels of entire transcripts were calculated by FPKM (fragments per kilobase million). Differentially expressed genes (DEGs) were selected with a fold change (FC) ≥ 1.5 and a false discovery rate (FDR) < 0.05 using the DESeq2_EBSeq. The Gene Ontology (GO) and Kyoto Encyclopedia of Genes and Genomes (KEGG) enrichment analyses were performed on identified DEGs using the Cluster Profiler R package.

### 5.7. Quantitative Real-Time PCR (qRT-PCR) Verification

In order to validate the transcriptome data, eight selected differentially expressed genes from the RNA-seq analysis [protein disulfide isomerase (*PDI*), peptidyl-prolyl cis-trans isomerase (*PPI*), ubiquitin-protein ligase E3 C (*UBE3C*), Heat shock protein 70 (*Hsp70*), zinc finger protein (*Msn2*), thiol-specific peroxiredoxin (*PRDX5*), C-5 sterol desaturase (*ERG3*), delta(9) fatty acid desaturase (*OLE1*),] were detected by quantitative real-time reverse transcriptase PCR (qRT-PCR). qRT-PCR data were normalized using the *P. pastoris* glyceraldehyde-3-phosphate dehydrogenase (*GAPDH*) coding gene as an internal reference gene. Primers were designed using Primer Premier 5.0 software based on the above sequences (Table S4). After 5 days of culture, the total RNA was extracted from each sample using the RNeasy Mini Kit (TransGen Biotech Co., Ltd., Beijing, China) and treated with DNase I to remove genomic DNA contamination, according to the manufacturer’s instructions. The concentrations of purified RNA were determined by measuring the absorbance of samples at 260 and 280 nm, and then first-strand cDNA was synthesized using the RevertAid First Strand cDNA Synthesis Kit (Thermo Fisher Scientific, Waltham, MA, USA). Internal reference gene and target genes were amplified using the CFX96 Touch Real-Time PCR Detection System (Bio-Rad, Hercules, CA, USA). The relative quantification of gene expression was estimated using the comparative 2^−∆∆Ct^ method, and all reactions were performed in triplicate.

### 5.8. Statistical Analysis

Each experiment was tested at least in triplicate, and all measurements were individually performed. Comparisons among groups were carried out using one-way analysis of variance (ANOVA) followed by Duncan’s multiple comparison tests. All data were reported as mean ± standard deviation, and *p* ≤ 0.05 was considered a significant difference. Statistical analysis was performed by SPSS 26.0 (IBM Corp., Armonk, NY, USA), and graphs were plotted using Origin 2021 (Origin Lab, Northampton, MA, USA).

## Figures and Tables

**Table 1:** Strains, plasmids, and primers used in this study.

Strains, Plasmids, and Primers	Description	References
Strains		
*E.coli* DH5α	General cloning host	Takara
*P. pastoris* GS115	Host strain (his4^−^, Mut^+^)	Invitrogen
GS115-FumDO	GS115 harboring pPIC9K-*FumDO*	This study
GS115-FumDM	GS115 harboring pPIC9K-*FumDM*	This study
GS115-FumDM/Bip	GS115-FumDM harboring pPICZA-*Bip*	This study
GS115-FumDM/HAC1	GS115-FumDM harboring pPICZA-*HAC1*	This study
GS115-FumDM/ERO1	GS115-FumDM harboring pPICZA-*ERO1*	This study
GS115-FumDM/CPR5	GS115-FumDM harboring pPICZA-*CPR5*	This study
GS115-FumDM/PDI	GS115-FumDM harboring pPICZA-*PDI*	This study
Plasmids		
pPIC9K	Secretion-expression vector	Invitrogen
pPIC9K-*FumDO*	pPIC9K derivative, carrying *FumDO* gene expression cassette	This study
pPIC9K-*FumDM*	pPIC9K derivative, carrying *FumDM* gene expression cassette	This study
pPICZA	Intracellular expression vector	Invitrogen
pPICZA-*Bip*	pPICZA derivative, carrying *Bip* gene expression cassette	This study
pPICZA-*HAC1*	pPICZA derivative, carrying *HAC1* gene expression cassette	This study
pPICZA-*ERO1*	pPICZA derivative, carrying *ERO1* gene expression cassette	This study
pPICZA-*CPR5*	pPICZA derivative, carrying *CPR5* gene expression cassette	This study
pPICZA-*PDI*	pPICZA derivative, carrying *PDI* gene expression cassette	This study
Primers		
*FumDO*-F	AGGCTGAAGCTTACGTAGAATTCATGGTGAAAGAGCACCAATGCCGTGG	*FumDO* amplification
*FumDO*-R	AAGGCGAATTAATTCGCGGCCGCCTATTTTGAGGGTTGGCAGGCTTTGC	*FumDO* amplification
*FumDM*-F	AGGCTGAAGCTTACGTAGAATTCATGGTTAAGGAACACCAATGTAGAGG	*FumDM* amplification
*FumDM*-R	AAGGCGAATTAATTCGCGGCCGCTTATTTAGAAGGTTGACAAGCCTTAG	*FumDM* amplification
*Bip*-F	ATTATTCGAAACGAGGAATTCGCCACCATGCTGTCGTTAAAACCATCTTG	*Bip* amplification
*Bip*-R	TGGCGGCCGCCGCGGCTCGAGGTCAACTCATCATGATCATAGTCATAG	*Bip* amplification
*HAC1*-F	CGGAATTCGCCACCATGCCCGTAGATTCTTCTCATAAG	*HAC1* amplification
*HAC1*-R	CCGCTCGAGGTCCTGATCGCTATGCATGTCAAC	*HAC1* amplification
*ERO1*-F	ATTATTCGAAACGAGGAATTCGCCACCATGAGGATAGTAAGGAGCGTAG	*ERO1* amplification
*ERO1*-R	TGGCGGCCGCCGCGGCTCGAGGTCAAGTCTACTCTATATGTGGTATCTC	*ERO1* amplification
*CPR5*-F	CGGAATTCGCCACCATGAAATTGTTGAACTTTCTGCTTAG	*CPR5* amplification
*CPR5*-R	CCGCTCGAGGTCAACTCATCTTTCACGACCTC	*CPR5* amplification
*PDI*-F	CGGAATTCGCCACCATGCAATTCAACTGGGATATTAAAAC	*PDI* amplification
*PDI*-R	CCGCTCGAGGTAAGCTCGTCGTGAGCGTCTG	*PDI* amplification

## Data Availability

The data that support the findings of this study are available from the corresponding author upon reasonable request.

## Supplementary Materials

The following supporting information can be downloaded at: https://www.mdpi.com/article/10.3390/toxins15020156/s1, Figure S1: Enzyme activities of recombinant strains GS115-FumDO and GS115-FumDM from 1st day to 5th day; Figure S2: Enzyme activities of recombinant strains co-expressing of molecular chaperones from 1st day to 5th day; Table S1: Summary of sequencing data quality; Table S2: Transcriptional up-regulated genes in PD group; Table S3: Transcriptional up-regulated genes in CP group; Table S4: Information table of qRT-PCR validation of differentially expressed genes.
