# Supplementary material for: Improved Production of Recombinant Carboxylesterase FumDM by Co-Expressing Molecular Chaperones in Pichia pastoris
Source: Toxins (Basel). 2023 Feb 14;15(2):156. doi: 10.3390/toxins15020156 (PMC9960120; doi:10.3390/toxins15020156)
Supplement: Supplementary file 1 [file toxins-15-00156-s001.zip › toxins-2198872-supplementary.pdf]

Supplementary Materials

Supplementary figures

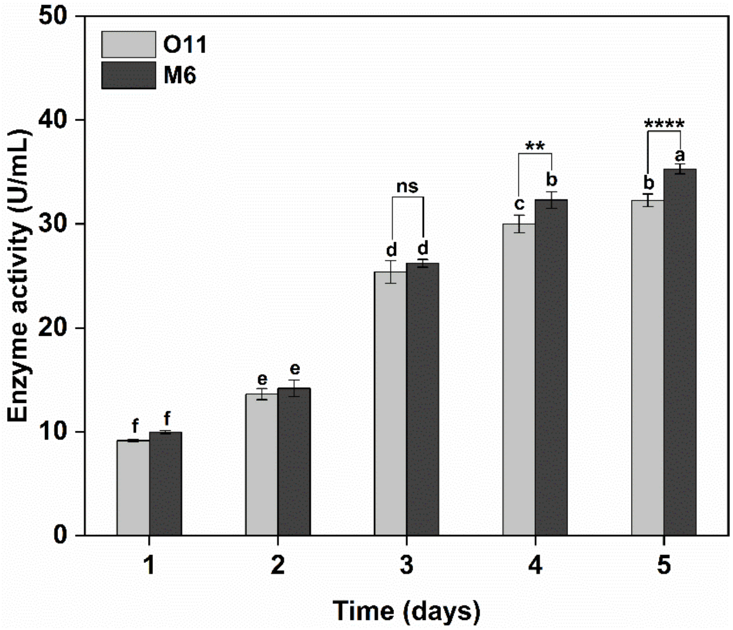

Figure S1. Enzyme activities of recombinant strains GS115-FumDO and GS115-FumDM from 1st day to 5th day.

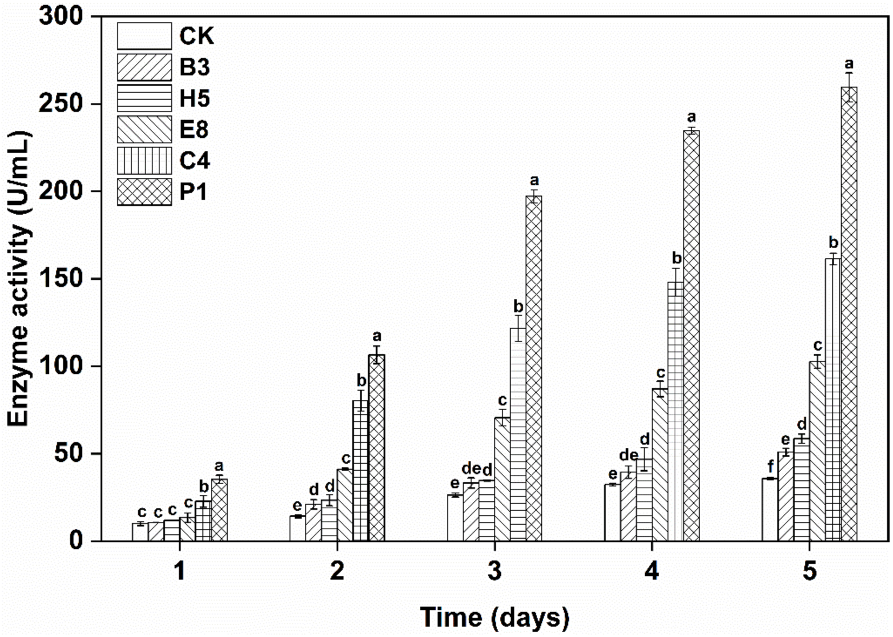

Figure S2. Enzyme activities of recombinant strains co-expressing of molecular chaperones from 1st day to 5th day.

Supplementary tables

Table S1. Summary of sequencing data quality.

Samples	Clean reads	Clean Bases	GC Content	%≥Q30
CK-1	20,203,621	6,049,706,214	43.98	94.94
CK-2	20,463,812	6,126,982,398	44.00	94.67
CK-3	25,828,645	7,731,982,152	44.23	95.62
CP-1	20,685,957	6,192,743,110	43.90	94.49
CP-2	20,431,706	6,119,436,324	43.95	94.32
CP-3	20,624,434	6,176,530,034	43.85	94.69
PD-1	20,744,156	6,212,332,468	43.96	94.06
PD-2	20,219,890	6,055,103,224	44.01	94.38
PD-3	19,384,889	5,804,669,746	43.95	93.59

Table S2. Transcriptional up-regulated genes in PD group.

**GO Class**	**GO ID**	**GO Term**	**Query ID**	**Description**	**Log_2FC**
MF	GO:0003756	protein disulfide isomerase activity	PAS_chr4_0844	Protein disulfide isomerase/*PDI*	2.19
MF	GO:0016491	oxidoreductase activity	PAS_chr3_0053	C-4 methyl sterol oxidase/*ERG25*	1.28
MF	GO:0003700	transcription factor activity	PAS_chr1-1_0381	transcriptional activator HAC1	1.01
MF	GO:0005524	ATP binding	PAS_chr3_0365	Mitochondrial matrix ATPase	1.23
MF	GO:0005515	protein binding	PAS_chr2-1_0490	glutathione S-transferase/*gst2*	1.02
MF	GO:0022857	transmembrane transporter activity	PAS_chr1-3_0017	High-affinity cysteine-specific transporter	0.96
MF	GO:0051082	unfolded protein binding	PAS_chr2-2_0151	Type II Hsp40 co-chaperone that interacts with the Hsp70 protein Ssa1	0.94
MF	GO:0003955	NAD(P)H dehydrogenase (quinone)	PAS_chr1-1_0449	NAD(P)H dehydrogenase (quinone)	1.29

		activity			
MF	GO:0016853	isomerase activity	PAS_chr4_0198	C-8 sterol isomerase/*ERG2*	1.07
BP	GO:0050896	response to stimulus	PAS_chr2-1_0723	Transcriptional activator, zinc finger protein/*Msn2*	1.49
BP	GO:0065007	biological regulation	PAS_chr3_0092	transcriptional enhancer factor	1.36
BP	GO:0008610	lipid biosynthetic process	PAS_chr1-4_0367	C-5 sterol desaturase/*ERG3*	1.52
BP	GO:0045332	phospholipid translocation	PAS_chr4_0430	Cell division control protein 50/*Cdc50*	1.12
BP	GO:0006457	protein folding	PAS_chr3_0230	Hsp70 protein involved in protein folding and the response to stress	1.15
BP	GO:0042026	protein refolding	PAS_chr4_0158	chaperonin GroEL, Tetradecameric mitochondrial chaperonin	1.15
BP	GO:0043248	proteasome assembly	PAS_chr4_0761	proteasome component ECM29	1.31
BP	GO:0006636	unsaturated fatty acid biosynthetic process	PAS_chr2-1_0072	Delta(9) fatty acid desaturase/*OLE1*	1.11
BP	GO:0051920	cell redox homeostasis	PAS_chr2-1_0502	Thiol-specific peroxiredoxin/*PRDX5*, reduces hydroperoxides to protect against oxidative damage	1.09
CC	GO:0005789	endoplasmic reticulum membrane	PAS_chr2-1_0038	ER transmembrane protein/*Bap31*	1.30
CC	GO:0016021	integral component of membrane	PAS_chr4_0784	aquaglyceroporin related protein, channel-like protein	0.99

Note: All the transcriptional up-regulated genes shown in the table were ranked by p-value with p ≤ 0.001.

Table S3. Transcriptional up-regulated genes in CP group.

GO Class	GO ID	GO Term	Query ID	Description	Log_2FC
MF	GO:0003755	peptidyl-prolyl cis-trans isomerase activity	PAS_chr1-1_0267	Peptidyl-prolyl cis-trans isomerase (cyclophilin)/*PPI*	5.73
MF	GO:0003955	NAD(P)H dehydrogenase (quinone) activity	PAS_chr1-1_0449	NAD(P)H dehydrogenase (quinone)	1.71
MF	GO:0003700	transcription factor activity	PAS_chr1-1_0381	transcriptional activator HAC1	0.60
MF	GO:0035091	phosphatidylinositol binding	PAS_chr2-1_0389	Sorting nexin	0.61
MF	GO:0016301	kinase activity	PAS_chr4_0678	Chitin synthase regulatory factor 3/*chr3*	0.65
MF	GO:0016853	isomerase activity	PAS_chr4_0198	C-8 sterol isomerase/*ERG2*	0.91
MF	GO:0008237	metallopeptidase activity	PAS_chr3_0517	peptidase family	0.62
MF	GO:0003676	nucleic acid binding	PAS_chr1-1_0386	Polynucleotide kinase 3 phosphatase	0.62
BP	GO:0034599	cellular response to oxidative stress	PAS_chr2-1_0640	nitroreductase/*HBN1*	1.35

BP	GO:0045332	phospholipid translocation	PAS_chr4_0430	Cell division control protein 50/*Cdc50*	0.70
BP	GO:0016570	histone modification	PAS_chr4_0902	RNA polymerase II-associated protein	0.61
BP	GO:0006635	fatty acid beta-oxidation	PAS_chr1-4_0538	Fatty-acyl coenzyme A oxidase	0.64
BP	GO:0016070	RNA metabolic process	PAS_chr1-3_0151	Nucleolar protein required for normal metabolism of the rRNA primary transcript	0.85
BP	GO:0006421	asparaginyl-tRNA aminoacylation	PAS_chr1-1_0392	Mitochondrial asparaginyl-tRNA synthetase	0.60
BP	GO:0045727	positive regulation of translation	PAS_chr1-4_0230	translation elongation factor GUF1	0.69
BP	GO:0000003	reproduction	PAS_chr2-2_0013	Subunit of a replication-pausing checkpoint complex (Tof1p-Mrc1p-Csm3p)	0.67
CC	GO:0005576	extracellular region	PAS_chr3_0076	Cysteine-rich secretory protein family	0.77
CC	GO:0005759	mitochondrial matrix	PAS_chr4_0755	Mitochondrial glycoprotein	0.68
CC	GO:0016020	membrane	PAS_chr1-1_0085	Membrane-associating domain	0.63
CC	GO:0005743	mitochondrial inner membrane	PAS_chr3_0880	Mitochondrial inner membrane protein required for normal mitochondrial morphology	0.65

Note: All the transcriptional up-regulated genes shown in the table were ranked by p-value with $p \leqslant 0.001$, except that p-value of mitochondrial inner membrane (GO: 0005743) was less than 0.05 ($p \leqslant 0.05$).

Table S4. Information table of qRT-PCR validation of differentially expressed genes

Gene Name	Encoding related proteins	Forward primer (5' to 3')	Reverse primer (5' to 3')
GAPDH	glyceraldehyde-3-phosphate dehydrogenase (GAP) gene	ATGGAGCAGTGATGACGACC	GCTGGTGTCGACTACGTCAT
PDI	protein disulfide isomerase	TCTTCAAGCTAGTCGGAAAG	CCTCATAAGCAGGAGCCATT
PPI	peptidyl-prolyl cis-trans isomerase (cyclophilin)	ACTTCAAACTCAAGCACGAT	ACGACATAGCCAAAGACAAC
Hsp70	heat shock 70kDa protein	GTCCATCAACCCAGACGAAG	TAGAGGAGCAACATCCAGCA
UBE3C	ubiquitin-protein ligase E3 C	CCTTCAACCCAGAGGGACAT	CACCGATTGCACAAGTAGCC
Msn2	zinc finger protein Msn2	CTTTGATTACGAACGGGATT	GAGGCTGATGCTGAGACG

PRDX5	thiol-specific peroxiredoxin	TGCCTGGAAGAAGACATTG	GTGGATGAAATCACCCTTA
ERG3	C-5 sterol desaturase	CAATCACCCAAGATACCTCA	TATCCACGCAACTCAATCAA
OLE1	delta(9) fatty acid desaturase	GGGGTGGATTTATCTACGG	GGTGTCCTCCTGTCATCG
